# Investigating the effect of health transformation plan on the public hospitals performance indicators; a case study from Iran

**DOI:** 10.1186/s12913-021-07164-y

**Published:** 2021-10-21

**Authors:** Mohammad Ranjbar, Mohammad Bazyar, Hassan Jafari, Mohsen Pakdaman, Vahid Pirasteh

**Affiliations:** 1 Health policy and management research center, Department of Health Services Management, School of public health, Shahid Sadoughi University of Medical Sciences, Yazd, Iran; 2 Department of Health Management and Economics, Ilam University of Medical Sciences, Ilam, Iran; 3 Health policy and management research center, School of public health, Shahid Sadoughi University of Medical Sciences, Yazd, Iran; 4 Department of Health Services Management, School of public health, Shahid Sadoughi University of Medical Sciences, Yazd, Iran

**Keywords:** Health Transformation Plan, Performance Indicators, Public Hospitals, Interrupted Time Series Model

## Abstract

**Background:**

Health systems need constant changes and reforms in their structure to adapt to changing conditions and meet the needs of society. One of the fundamental changes in the health system of Iran is the health transformation plan (HTP), the effects of which must be examined from different aspects. Therefore, the purpose of this study is to investigate the effect of HTP on the performance indicators of public hospitals in Yazd city, Iran.

**Methods:**

This cross-sectional study was carried out in all public hospitals in city of Yazd. Six performance indicators were examined monthly and in two time periods of 12 months before and 12 months after the implementation of Health Transformation Plan (HTP). The data was analyzed by SPSS software program version 22, using the paired T-test, and the Interrupted Time Series (ITS) model.

**Findings:**

Findings showed that the performance indicators of the studied hospitals have improved after the implementation of the HTP. According to the ITS model, the implementation of HTP did not have a significant effect on the level and trend of the bed rotation distance, average length of stay and the ratio of surgical operations to bed indicators. However, it had a statistically significant effect on the level and trend of mortality and hospitalization rates. Moreover, the implementation of HTP had a significant effect on the level of the bed occupancy rate, but did not have a significant effect on the trend of this indicator.

**Conclusion:**

Based on the research findings, all the selected indicators changed to some extent after the implementation of HTP, which showed the effect of this plan on the performance of hospitals. However, not all indicators were statistically significant as the findings sub-section revealed.

## Background

Over the past decades, several countries including Turkey, Thailand, China, South Korea, Ghana and Mexico have introduced reforms aimed at improving financial access to medical services and increasing quality and equity in the health system[1–5]. For example, in 2003, major reforms were implemented in the health system in Turkey, aiming to boost primary health care services, integrate health financing and reorganize the health system which were successful in improving health equity and access to health services for the underprivileged populations[6].

In Iran, several major health reforms have been implemented over the last four decades aiming to improve the health system performance[7–12]. These reforms include the formation of the Ministry of Health and Medical Education in 1985 to solve problems related to training and supplying of health workforces; establishment of vast District Health Network in 1985 to provide affordable primary health care services for the whole population especially in deprived regions and rural areas; establishment of Universal health Insurance Organization in 1994 to achieve universal health insurance coverage; and implementation of Family Physician Program in the rural areas in 2005 to organize health system [10–16].

Although these reforms made considerable achievements in the health system including improvement in public health indicators and increase in human resources and physical and financial resources, there were still several main problems such as high out-of-pocket payments, inequity in access to and utilization of health care services among different groups of the insured population, low quality of health services in the public health sector, and no health insurance coverage for a part of the population [10, 11, 17]. To address these chronic problems, in line with the changes implemented in other countries, the Ministry of Health and Medical Education of Iran, in order to achieve the vision of 2025, has designed a comprehensive transformation plan in the health system, so-called Health Transformation Plan (HTP), which has been implemented since May 5, 2014, and is now ongoing[18]. The HTP began with three approaches (financial protection for the patients, improving equity in access to health services, and improving the quality of services) consisting of seven programs[11, 19]. As a result of HTP, several main changes occurred in Iran, for instance, government spending on health increased unprecedentedly, health insurance coverage extended to all populations and about 11 million people were covered, medical tariffs were revised and about 1700 medical services were added into the basic benefit package, new hospitals and public health facilities were created in deprived regions, many hospitals and public health facilities were renovated, hospital beds increased, and more physicians and health workers, such as nurses and midwives were recruited in remote areas[11, 20]. Table 1 shows some the main demographic and health system indicators in Iran[10].

**Table 1:** main demographic and health system indicators in Iran (2016)

Indicator	2016
Total population	79,926,270
Family size	3.3
Urban population (%)	74
Rural population (%)	24.9
Literacy rate (%)	87.6
Hospital beds per 1000 population	1.72 (2017)
Overall bed occupancy rate in public hospitals (days)	75 (2017)
Mean length of stay in public hospitals (days)	2.78 (2017)
Population/physician ratio	900 (2017)

Table 2 also presents the number of hospitals in Iran affiliated with different organizations in 2018[10].

**Table 2:** Number of teaching and non-teaching hospitals and their staff in Iran (2018)

Organization	No of hospitals	Beds	Number of staff
**The Universities of Medical Sciences (UMS) affiliated to the MoHME**	749	90,003	261,497
**The Private sector**	166	16,626	70,966
**The SSO**	73	10,853	32,984
**The Military**	32	2190	2559
**Charity Organizations**	37	4198	15,732
**Islamic Revolutionary Guard Corps**	17	2483	1060
**The Iranian National Oil Corporation (NIOC)**	9	967	5195
**Other sectors**	34	3592	12,130
**Total**	1117	130,912	402,123

As a result of the above initiatives introduced in the health system, it is not surprising to see an increase in access and utilization of health care services soon after the implementation of HTP. The impact of HTP on different aspects of the Iranian health system has been studied by different studies over the last years. For instance, according to official statistics, the cost of out-of-pocket payments in Iran has been declining since the beginning of the health system transformation plan, from 58 % to 2011 to 49 % in 2013, 40 % in 2014, and finally 32.46 % in 2017[17, 21]. As such, the purpose of the HTP is to improve people’s health, reduce out-of-pocket payments and develop health indicators[22]. HTP increased access to outpatient and inpatient health care services, for instance in 2015 and for patients covered by the Iran Health Insurance Organization (one of the three main social health insurance funds in Iran) alone, utilization of inpatient services and average hospitalization costs increased by 6 % and by more than 73 % respectively during the years 2012-2016[17]. Several studies in Iran have been conducted to investigate whether the HTP has improved the hospital performance indicators especially in terms of using medical resources efficiently[23–25]. The most important functional indicators to measure the efficiency of hospitals are Bed Occupancy Rate (BOR), Bed Rotation Distance (BRD), Average Length of Stay (ALS), the ratio of surgeries to the bed, the rate of hospitalization per bed, and the mortality rate [26, 27].

Studies by Dadgar, Rezaei, Mousavi Rigi, Ghazizadeh, Yusefi, and Hashemian separately examined the effect of health transformation plan on performance indicators in different hospitals in Iran and concluded that the implementation of HTP has led to a significant increase in inpatient health services utilization and in turn in the performance indicators of hospitals[22, 26, 28–31]. Although several studies have been conducted in different parts of Iran regarding the impact of the implementation of HTP on the performance indicators of hospitals, so far no study has been done in this regard in public hospitals of Yazd Province. As a result of high-quality health services in Yazd city, Yazd health centers are considered as referral centers for patients from other provinces needing outpatient and inpatient health services. Many people from the southern and southeastern regions of Iran come to these hospitals for their health needs.

Yazd Province is located in the center of Iran. At the 2016 census, its population was about 1.138.000. Shahid Sadoughi University of Medical Sciences administers 13 public hospitals and over 90 clinics throughout the city and province of Yazd. There are also 8 other kinds of hospitals including private hospitals and hospitals affiliated with Social Security Organization, Islamic Azad University etc. There were 292 general physicians, 843 specialists, 1000 dentists, 130 pharmacists, and 680 nurses in Yazd province in 2017. The number of active hospital beds in the Yazd province in 2017 was 2664, of which 1671 beds were public. According to the report by the treatment deputy of Shahid Sadoughi University of Medical Sciences, more than 50 % of hospitalizations in Yazd province are from other provinces[32, 33]. So this study was conducted to investigate the effect of HTP on the level and trend of performance indicators of the public hospitals in Yazd city.

## Methods

### Selection of hospitals and performance indicators

The present study is analytical research using the interrupted time series method to investigate the impact of the implementation of HTP on the trend of changes in the performance indicators of public hospitals in the city of Yazd. There are ten hospitals in Yazd city, including four public hospitals, four private hospitals, one hospital affiliated with Social Security Organization as one of the main social basic health insurance schemes, and one hospital for Islamic Azad University. Three of these public hospitals are general (named Shahid Sadoughi, Rahnamoun, and Afshar) and one of them is a specialized hospital. These general hospitals are big and generally similar to each other. So we expected to see similar changes in these hospitals in the selected indicators. As HTP was implemented in the public health sector only, we chose these three hospitals for the purpose of the study. We focused on the hospital performance indicators especially in terms of efficiency including BOR, BRD, ALS, the ratio of surgeries to the bed, the rate of hospitalization per bed, and the mortality rate. We chose these indicators as they are collected routinely by the medical statistics units in the hospitals as the main indicators to measure efficiency in hospitals and we expected to see rapid changes in these indicators as a result of HTP implementation.

### Data collection and duration of the study

Data related to hospital performance indicators were collected in the form of Excel sheets and on a monthly basis for the two periods of 12 months before (from April 21, 2013, to April 20, 2014) and 12 months after (from May 22, 2014, to May 21, 2015) the implementation of HTP. The process of data collection was done in 2019. Data were provided by the medical statistics and nursing units of studied hospitals and also by the deputy of treatment of the Yazd University of Medical Sciences. Data were analyzed by SPSS software version 22. We combined the data from the three hospitals for all the analyses.

### Statistical methods

To check the difference in the annual average of each indicator for the year before and after the HTP, the paired t-test was applied. Then, using the interrupted time series analysis method, the data in the two periods before and after HTP were considered as an interrupted time series and the effects of the transformation plan on both the level of indicators and the trend of changes of the indicators after the plan were examined. In the interrupted time series model (ITS), two variables show the effect of a policy or intervention; the first one is the level which determines the immediate effect of the intervention and the second is the trend variable that indicates the long-term effect of the intervention. The passage of time in the present study refers to the months that have passed since the implementation of the HTP, in which we considered 12 months after the transformation plan in addition to 12 months before the plan (although May is not included in the analysis). ITS can reveal the probable changes (immediate and short-term changes) in the level of indicators at the beginning of the project, i.e. in the 13th month and also how the trend of each indicator has changed over time (month by month). To estimate the ITS model, according to the use of time-series data, first to prevent the false regression estimation of static data, the data of the indicators were examined using the root test of the Dickey-Fuller unit. Accordingly, the null hypothesis stating that there is a single root for all indicators is rejected (*p* <0.05) and the time series is static for all indicators. Then, for each of the indicators, the following regression pattern is estimated:

Y = β_0_ + β_1_T + β_2_X + β_3_XT + ε.

Y represents the value of each indicator per month. T, X, XT and ε indicate time, interference, the interaction of time and interference, and part of the error, respectively. Since the month of the intervention (here HTP), X1, is an imaginary variable, takes the value of 1 and before that the value of zero. β_0_, β_1_, β_2_, and β_3_ indicate a constant value, time trend without considering an intervention, immediate effects of the intervention on the level of selected indicators and continuous effect of the intervention on the trend of indicators, respectively.

## Results

Here we present the findings of the study and the effect of HTP on the selected indicators according to the paired t-tests and ITS analysis. Table 3 shows that the total number of active beds for one of the hospitals, and the total number of hospitalizations, has increased during the first year after the implementation of the HTP.

**Table 3:** Total number of fixed and active beds and the total number of hospitalizations in the public hospitals in the city of Yazd, Iran, during 12 months before and 12 months after the Health Transformation Plan (2013-2015)

Variable Hospital	Number of fixed beds		Number of active beds		Total number of hospitalization	
	12 months before the HTP	12 months after the HTP	12 months before the HTP	12 months after the HTP	12 months before the HTP	12 months after the HTP
Shahid Sadoughi	582	582	387	428	30,705	34,540
Rahnamoun	200	200	152	152	6857	11,277
Afshar	220	220	155	144	10,572	14,952
Total	1002	1002	694	724	48,134	60,769

Table 4 shows the results of running paired t-tests for comparing the mean of performance indicators in 12 months before and after the implementation of the HTP. According to the findings, the mean of “bed occupancy rate”, “hospitalization rate” and “the ratio of surgical operations to bed” has increased significantly (see p-values in Table 4) and at the same time “bed rotation distance”, “average patient stay” and “mortality rate” has decreased after the implementation of the transformation plan. All of these changes show that efficiency in using the beds has increased after the HTP.

**Table 4:** Mean and standard deviation of performance indicators of public hospitals in the city of Yazd, Iran, before and after the implementation of the Health Transformation Plan (2013-2015)

Indicator	Mean		SD		p-value^*^
	12 months before the HTP	12 months after the HTP	12 months before the HTP	12 months after the HTP	
Bed Occupancy Rate (%)	71.89	79.86	7.52	6.84	0.01
Bed Rotation Distance (day)	1.56	0.86	0.64	0.43	0.02
Average Length of Stay (day)	3.89	3.44	0.39	0.40	0.01
Mortality rate (per 1000 discharged patients)	25.19	24.22	13.23	15.36	0.74
Hospitalization rate (per month)	1420.44	1688.03	837.43	886.68	0.19
The ratio of surgical operations to bed	3.11	5.25	1.02	2.38	0.01

* *P*- values and estimations were driven by paired t-tests

**Fig. 1:**
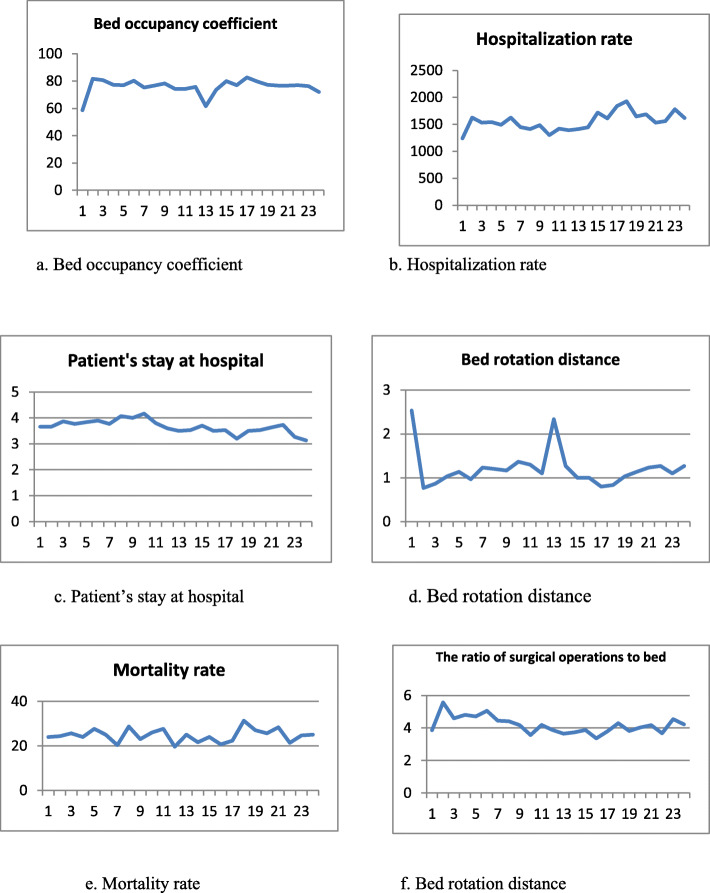
The trend of changes in the performance indicators of public hospitals in Yazd during the research period (from April 21, 2013, till May 21, 2015).

Table 5 shows the results of the interrupted time series analysis for the performance indicators of the studied hospitals. According to Table 5, the coefficient of the level indicates the immediate effect of the HTP on each indicator, and the coefficient of the trend indicates the continuous effect of the HTP on the trend of each of the indicators. According to the ITS test, the HTP had an immediate effect on increasing the level of the number of hospitalizations and bed occupancy rate but the trend of bed occupancy rate had a declining slope (β_3=_ - 0.006) over time. According to the ITS analysis, mortality rate decreased after the HTP but experienced an increasing slope over time. ITS did not show significant difference for the level and trend of other indicators including bed rotation distance, average length of stay at hospital, and the ratio of surgical operations to bed. The trend of changes in the all indicators over time is shown in Fig. 1.

**Table 5:** Evaluation results of interrupted time series model for the effect of health transformation plan on the performance indicators of public hospitals in the city of Yazd, Iran (2013-2015)

Indicator title	Variable	Coefficient	p-value
Bed occupancy rate	Level change due to intervention (β2)* Procedure change due to intervention (β3)*	4.45 - 0.006	0.02 0.94
Bed rotation distance	Level change due to intervention (β2) Procedure change due to intervention (β3)	-0.30 -0.007	0.88 0.92
Average length of stay at hospital	Level change due to intervention (β2) Procedure change due to intervention (β3)	0.29 -0.06	0.77 0.40
Mortality rate	Level change due to intervention (β2) Procedure change due to intervention (β3)	-4.83 0.26	0.01 0.01
Hospitalization rate	Level change due to intervention (β2) Procedure change due to intervention (β3)	136.5 31.41	0.01 0.01
The ratio of surgical operations to bed	Level change due to intervention (β2) Procedure change due to intervention (β3)	1.52 0.07	0.36 0.12

*(β2) indicates immediate effects of the intervention on the level of selected indicators

* (β3) indicates continuous effect of the intervention on the trend of studied indicators

## Discussion

The findings showed that the number of hospitalizations increased after the implementation of the HTP, although this was not statistically significant. Another study by Ghazizadeh also confirmed the positive nature of the HTP and the increase in the number of hospitalizations in East Azerbaijan, Iran[26]. The reason for this increase can be attributed to the goals of the HTP, including reducing patients’ out-of-pocket payments and financially protecting patients against expensive and hard-to-cure diseases and also extending the free health insurance coverage for groups of population without coverage[29]. According to the Ministry of Health and Medical Education, just within the first year after the implementation of the HTP, patients coinsurance rates decreased from 37 to 4.5 % on average for the inpatient health services in the public health sector, which in turn increases people’s access to health services, especially for the poor. This led to an increase in hospital admissions[26]. Shen et al. examined the effects of the health transformation plan on the performance of hospitals in China from 2001 to 2005 and concluded that the new health reform policy had positive effects on reducing patients’ economic burden [34].

Study showed the bed rotation distance after the implementation of the transformation plan has been decreasing. In a study by Faridfar and et al., they concluded that HTP had a growing positive impact on hospital productivity [35]. The reason can be attributed to the increase of hospitalizations and also bed occupancy rate after the implementation of the HTP (46). Additionally, according to Hashemian’s study, the average of this indicator in university centers of Isfahan province has increased from 70.2 times in 2012 to 71.2 in 2013, 73.9 in 2014 and 76.4 times in 2015[31].

ITS analysis also showed that as a result of HTP, the bed occupancy rate increased, but the trend or slope of the indicator has decreased over time. Rezaei also confirmed the positive implementation of the HTP and the increase of this indicator in Hamedan’s hospitals, Iran [22]. Yasar also showed that the implementation of the transformation plan in the Turkish health system has increased hospital bed occupancy rate [22]. The following reasons could have been influencing in increasing the bed occupancy indicator including: reducing patient payments, retaining physicians in disadvantaged areas, the presence of specialist physicians in public hospitals, improving the quality of hospital services, improving hospital hoteling services, and financial protection against high health care expenditures[36] (49). According to Yusefi’s study, the average of this indicator in public hospitals in Shiraz, Iran, has increased by 8 % and increased from 72.7 % (2013) to 80.55 % (2015) [30]. Similar studies by Rezaei, Dadgar, Zarei, and Yusefi also revealed the same results [22, 23, 29, 30].

The ratio of surgeries to bed has increased significantly after the implementation of the HTP. This increase indicates that the treatment of patients who needed surgery was performed on time by specialist physicians present at the hospitals. Moreover, increasing the quality of hospital visits and hoteling services after the implementation of HTP can justify this increase[22]. In a study that examined the program of the HTP in Turkey, the effect of the plan on increasing the number of surgeries was significant[37]. Additionally, according to Yusefi’s study, the average monthly number of emergency surgeries in public hospitals in Shiraz has reached from 189.18 (2013) to 208.43 (2015) and at the same time the average of elective surgeries increased from 517.95 to 637.71 [30].

According to the findings, the HTP increased the average length of stay of the patients in hospitals, although it was not statistically significant. This finding is inconsistent with the results of Rezaei’s study carried out in Hamadan hospitals [22]. Numerous studies in Isfahan hospitals have shown that this plan has increased the length of stay of patients. This led to a lack of beds for other patients. One of the reasons for the increase in the length of stay in Lorestan hospitals after the HTP was the increase in the number of expensive and more complicated surgeries, which required longer stay in hospital [29]. Moreover, according to Hashemian’s study, the average patient stay for all hospitals studied between 2012 and 2015 was 2.8, 3, 2.8 and 2.9, respectively [31].

In the present study, the mortality rate has significantly decreased after the implementation of the health transformation plan. Of course, it should be noted that various factors such as time, place, the number and experience of health workers, the sufficiency and quality of medical equipment and supplies and etc. can affect the mortality rate and one cannot judge the performance of hospitals just base on the number of deaths [38].

## Limitations of the study

We just investigated the effect of HTP as the main intervention on the hospital performance indicators, while there may be other factors affecting the trend of hospital indicators which have not been taken into account in this research. Another limitation was that we just focused on data from three public hospitals in Yazd city and excluded other public and private hospitals in the whole of Yazd province which limits comparing the changes of studied indicators in these hospitals. We also combined the data for all three hospitals for analysis which ignores the probable heterogeneity among the studied hospitals which can limit the generalizability of the findings.

## Conclusions

Based on the research findings, all the selected indicators changed to some extent after the implementation of HTP, which showed the effect of this plan on the performance of the hospitals. However, not all indicators were statistically significant as the findings sub-section revealed. Improvement of the hospitals’ performance indicators as a result of HTP’ initiatives indicates indirectly that HTP has increased the access of people to the health care services. This might have increased equity in health services utilization by improving the access of those people who suffered from lack of health insurance coverage or had to face high coinsurance and copayment rates for using health services in hospitals. It is necessary to investigate the effects of HTP on the performance of other kinds of hospitals such as private hospitals and also the utilization of health care services by different groups of population. Such investigations can help policy makers to make necessary modifications in the programs in order to make them more accessible.

## Data Availability

The datasets generated and analysed during the current study are not publicly available as it is not allowed by the Research Deputy of Shahid Sadoughi University of Medical Sciences but are available from the corresponding author on reasonable request.

## Abbreviations

HTP: health transformation plan
BOR: Bed Occupancy Rate
BRD: Bed Rotation Distance
ALS: Average Length of Stay
